# High prevalence of late presentation with advanced HIV disease and its predictors among newly diagnosed patients in Kumasi, Ghana

**DOI:** 10.1186/s12879-024-09682-6

**Published:** 2024-07-31

**Authors:** Samuel Asamoah Sakyi, Samuel Kwarteng, Ebenezer Senu, Alfred Effah, Stephen Opoku, Success Acheampomaa Oppong, Kingsley Takyi Yeboah, Solomon Abutiate, Augustina Lamptey, Mohammed Arafat, Festus Nana Afari-Gyan, Samuel Kekeli Agordzo, Oscar Simon Olympio Mensah, Emmauel Owusu, Tonnies Abeku Buckman, Benjamin Amoani, Anthony Kwame Enimil

**Affiliations:** 1 Department of Molecular Medicine, School of Medicine and Dentistry, Kwame Nkrumah University of Science and Technology, Kumasi, Ashanti region Ghana; 2 Department of Medical Diagnostics, Faculty of Allied Health Sciences, Kwame Nkrumah University of Science and Technology, Kumasi, Ashanti region Ghana; 3 Department of Medical Microbiology, College of Health Sciences, University of Ghana Medical School, Accra, Greater Accra region Ghana; 4 Department of Medical Laboratory Sciences, KAAF University College, Buduburam, Accra, Greater Accra region Ghana; 5 Department of Biomedical Science, School of Allied Health Sciences, University of Cape Coast, Cape Coast, Central region, Ghana; 6 Pediatric Infectious Disease Unit, Child Health Directorate, Komfo Anokye Teaching Hospital, Kumasi, Ashanti region Ghana

**Keywords:** Latent HIV, HIV/AIDS, Advance diseases, Self-testing

## Abstract

**Background:**

Late presentation with advanced HIV disease (LP-AHD) remains a significant challenge to Human Immunodeficiency Virus (HIV) care, contributing to increased morbidity, mortality, and healthcare costs. Despite global efforts to enhance early diagnosis, a considerable proportion of individuals with HIV infection are unaware of being infected and therefore present late for HIV care. For the first time in Ghana, this study assessed the prevalence of LP-AHD and associated factors among people diagnosed with HIV (PDWH).

**Method:**

This bi-center retrospective cross-sectional study included 315 PDWH at the Aniniwah Medical Centre and Komfo Anokye Teaching Hospital, both in Kumasi, Ghana. A well-structured questionnaire was used to collect data on sociodemographic, clinical, lifestyle and psychosocial factors from the study participants. Statistical analyses were done in SPSS version 26.0 and GraphPad Prism version 8.0 at significant *p*-value of < 0.05 and 95% confidence interval. Predictors of LP-AHD were assessed using binary logistic regression models.

**Results:**

This study observed that, 90 out of the 315 study PDWH (28.6%) reported late with advanced HIV disease (AHD). Participants within the age group of 36–45 years (adjusted Odds Ratio [aOR]: 0.32, 95% CI: 0.14–0.69; *p* = 0.004) showed a significantly decreased likelihood of LP-AHD. However, participants who perceived cost of HIV care to be high (aOR: 7.04, 95% CI: 1.31–37.91; *p* = 0.023), who were diagnosed based on clinical suspicion (aOR: 13.86, 95 CI: 1.83–104.80; *p* = 0.011), and missed opportunities for early diagnosis by clinicians (aOR: 2.47, 95% CI: 1.30–4.74; *p* = 0.006) were significantly associated with increased likelihood of LP-AHD.

**Conclusion:**

The prevalence of LP-AHD among PDWH in Ghana is high. Efforts to improve early initiation of HIV/AIDS care should focus on factors such as the high perceived costs of HIV care, diagnosis based on clinical suspicion, and missed opportunities for early diagnosis by physicians.

## Introduction

Acquired Immune Deficiency Syndrome (AIDS) caused by Human Immunodeficiency Virus (HIV), remains a significant global health challenge despite efforts by international and local initiatives to address the epidemic [1]. At the end of 2022, an estimated 39 million people worldwide were living with HIV [2]. Africa remains the most affected, with nearly 1 in every 25 adults (3.4%) living with HIV and accounting for more than two-thirds of the people living with HIV worldwide [3]. Ghana has HIV prevalence of 1.7%, affecting 334,713 people and accounting for over fourteen thousand annual deaths [4]. HIV testing has been a gateway to HIV care, however coverage with HIV testing services is not adequate [5]. Despite the availability and accessibility of HIV testing, people continue to test late in the course of HIV infection [6].

In 2015, WHO recommended that all people living with HIV start ART (Antiretroviral therapy) irrespective of clinical or immune status [7]. However, people living with HIV continue to present to care late and with advanced disease [7]. Late presentation with Advanced HIV disease (LP-AHD) is defined as an individual presenting with a CD4 + count lower than 200 cells/µL or at WHO clinical stage-3 or stage-4 [8]. LP-AHD can lead to serious outcomes, such as increased mortality [9, 10], development of opportunistic infections [11], increased risk of drug resistance to antiretroviral therapies (ART) [12], high healthcare costs [12], and increased transmission due to ignorance of infection status [13].

Multiple sociodemographic, psychosocial, and structural risk factors at the patient and provider levels have been found to be associated with LP-AHD. Fear of HIV-related stigma [14] and discrimination [15], poor social support [14], and low risk perception [13] are among the common patient-related factors that discourage people from seeking timely testing. Providers have described inadequate time and resources [16], the burdensome counseling and consent process [17], and the provider’s perceived low risk of transmission [18], as barriers to providing HIV testing.

Despite the challenges LP-AHD poses to HIV care, there is a paucity of data on the prevalence and factors associated with LP-AHD. There is no study that has been done to assess the scope of LP-AHD in the Ghanaian context. For the first time, we determined the prevalence and predictors of LP-AHD among newly diagnosed HIV/AIDS subjects in Ghana.

## Materials and methods

### Study design and study site

A retrospective cross-sectional study was conducted at HIV clinics of Komfo Anokye Teaching Hospital (KATH) and Aniniwah Medical Centre (AMC) in the Ashanti Region of Ghana. KATH and AMC are located in Kumasi, the capital of the Ashanti region of Ghana and have a well-resourced HIV care clinics making it suitable for the successful implementation of the study.

### Study population

This study recruited newly diagnosed individuals confirmed of HIV/AIDS by clinicians using the standard diagnoses protocols.

### Inclusion criteria

Participants eligible for inclusion in this study were adults aged 18 years and older with a newly confirmed diagnosis of HIV infection based on standardized diagnostic criteria. In addition, these participants were willing to share relevant information for the research study. Also, these individuals had complete and valid data for the study variables, which encompass sociodemographic, clinical, and psychosocial factors considered in the study.

### Exclusion criteria

Individuals below the age of 18 are excluded, as well as those with incomplete or missing data related to crucial study variables encompassing sociodemographic, clinical, and psychosocial factors. Additionally, individuals who decline informed consent or express unwillingness to share pertinent information for research purposes were not considered.

### Sample size calculation and sampling technique

The sample size was calculated using the Cochrane formula: $\:n=\:\frac{{z}^{2}pq}{{e}^{2}}$ ; where: n is the minimum sample size, *p* is the prevalence of HIV in Ghana = 1.7% [6], q = 1-p, (98.3), z = z value at 95% confidence (1.96), and e is the margin of error (0.05). Hence a minimum of twenty-six (26) participants were required for the study. To increase statistical power, 315 people diagnosed of HIV/AIDS were included for the study. The participants included 252 from the Komfo Anokye Teaching Hospital (KATH) and 63 from the Aniniwah Medical Centre, both within the Ashanti Region of Ghana.

### Ethical consideration

Ethical approval was sought from the Committee on Human Research, Publication, and Ethics at the School of Medicine and Dentistry of the Kwame Nkrumah University of Science and Technology (KNUST) (CHRPE/AP/385/23). Written informed consent was sought from each participant before the data collection of the study. Participation was voluntary and participants were allowed to opt out any time on their personal reasons.

### Data collection

Data for this study were collected using a structured questionnaire comprising four main sections: Sociodemographic, Lifestyle, Clinical Factors, and Psychosocial Factors. The questionnaire was designed to gather comprehensive information pertaining to the study’s objectives. Data were collected through face-to-face interviews conducted by trained research assistants. Participants’ responses were recorded as per their answers to the structured questions in the questionnaire. Participants were stratified into late presenters and non-late presenter based on the WHO HIV staging system.

### Definition of dependent variables

**Participant with LP-AHD** – People confirmed with HIV/AIDS at WHO stage 3 or 4 at the time of diagnosis.

**Participant without LP-AHD*–*** People confirmed with HIV/AIDS at WHO stage 1 or 2 at the time of diagnosis.

### Data management and statistical analyses

Data analysis was performed using Statistical Package for the Social Sciences 26.0 software (SPSS, Inc.; Chicago, IL, USA) and GraphPad Prism version 8.0. Categorical variables were presented as frequencies and percentages. A simple bar chart was used to illustrate the prevalence of LP-AHD among study participants. Pearson chi-square test or Fischer exact test was conducted to ascertain the relationship between LP-AHD and the study variables. Variables were also tested in the Univariate logistic regression prediction model and significant variables from the univariate and potential cofounders were tested in the multivariate logistic regression prediction model to assess the independent predictors of LP-AHD. *p*-value of less than 0.05 and 95% confidence interval were considered statistically significant.

## Results

### Sociodemographic characteristics of study participants

Of the 315 participants enrolled in the study, one-third (33.3%) were within the ages of 46-55years, one-quarter (25.4%) were within the age range of 36–45 years with the least percentage of age range being 18–35 years (13.0%). Most of the participant in the study were females (85.7%) with males accounting for 14.3% of the population. Considering marital status, 37.5% were married with 17.5% being single. Also, a higher percentage of the participants had 2–3 children (44.3%) and Junior High School education (39.0%). Majority of the enrolled participants lived in urban areas (85.7%), were Christians (87.6%) and Akans (81.6%). With respect to employment status, 7.3% were employees whilst 63.5% were self-employed. Moreover, 47.5% of the participants earned <$\not C$500 and 45.0% took 31–60 min to reach the clinic on visit days **(**Table 1**).**

**Table 1:** Sociodemographic characteristics of study participants

Variable	Frequency (*n* = 315)	Percentage (%)
**Age Group (Years)**		
18–35	41	13.0
36–45	80	25.4
46–55	105	33.3
56–90	89	28.3
**Gender**		
Female	270	85.7
Male	45	14.3
**Marital Status**		
Single	55	17.5
Divorced	61	19.4
Widowed	81	25.7
Married	118	37.5
**Number of Children**		
0–1	73	23.2
2–3	139	44.3
≥ 4	102	32.5
**Educational Level**		
No formal	64	20.3
Primary	63	20.0
JHS	123	39.0
SHS	49	15.6
Tertiary	16	5.1
**Residence**		
Rural	45	14.3
Urban	270	85.7
**Employment Status**		
Not employed	92	29.2
Self employed	200	63.5
Employed	23	7.3
**Ethnicity**		
Akan	257	81.6
Northerners	46	14.6
Others	12	3.8
**Religion**		
Christianity	275	87.6
Muslims	36	11.5
Others	3	1.0
**Monthly Income (Gh**$\not C$)		
< 500	149	47.5
500–1000	107	34.1
> 1000	58	18.5
**Time taken to reach hospital (minutes)**		
5–30	74	23.6
31–60	141	45.0
> 60	98	31.3

Data presented as frequency and percentage.

### Prevalence of LP-AHD

**Fig. 1:**
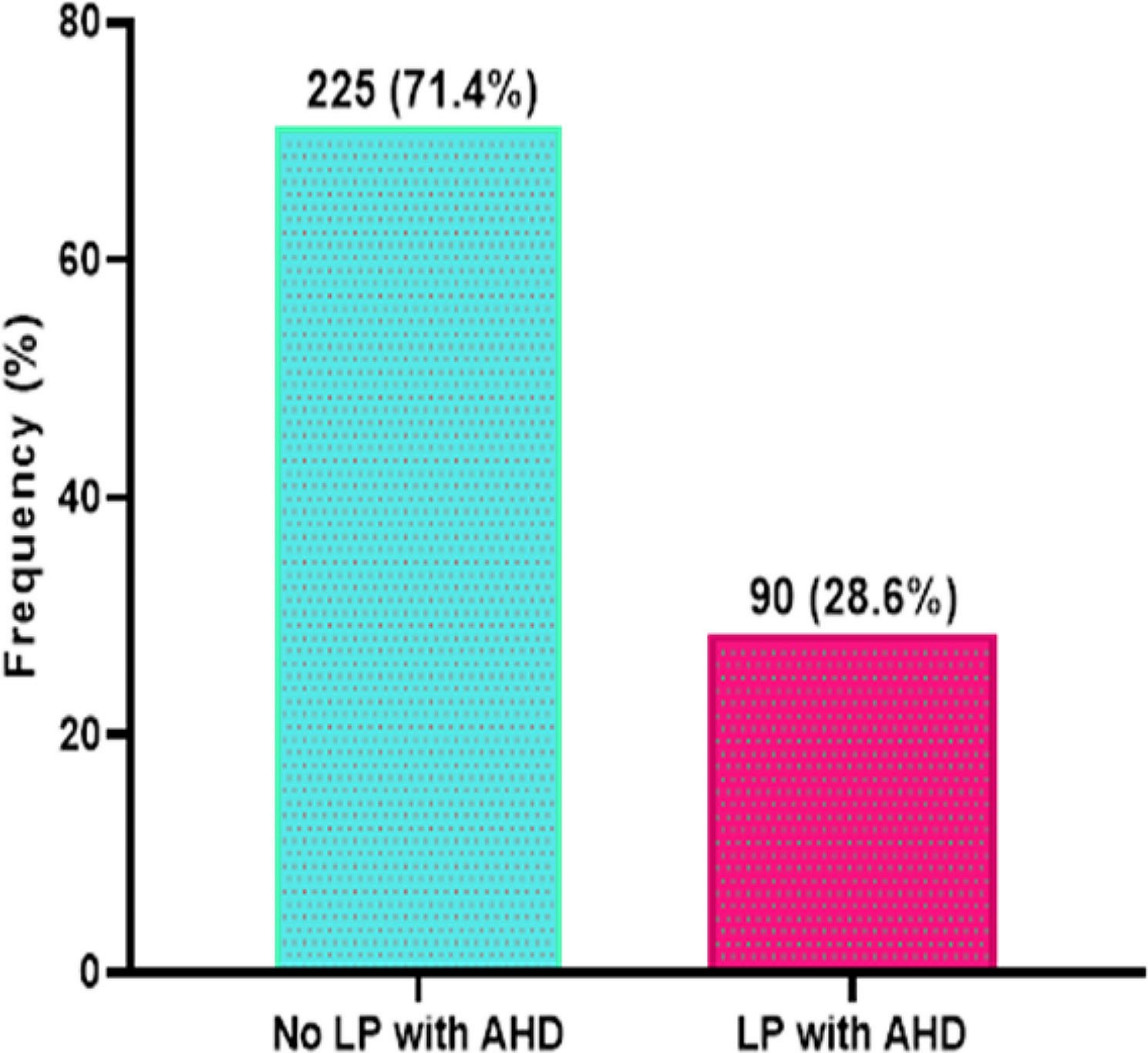
Prevalence of late presentation with advanced HIV disease (LP-AHD)

The study found that the prevalence of LP-AHD among the study participants was 28.6% which accounted for 90 of the participants. 225 of the study participants did not present late (71.4%) (Fig. 1**).**

### Sociodemographic factors associated with late presentation with advanced HIV disease (LP-AHD)

The age of the study participants showed a significant association with LP-AHD (*p* = 0.002). On the contrary, gender (*p* = 0.140), educational level (*p* = 0.480), marital status (*p* = 0.257), number of children (*p* = 0.290), educational level (*p* = 0.480), residence (*p* = 0.067), employment status (*p* = 0.183), ethnicity (*p* = 0.933), religion (*p* = 0.678), monthly income (*p* = 0.784), and time taken to reach the clinic (*p* = 0.928) did not exhibit statistically significant associations with LP-AHD **(**Table 2**).**

**Table 2:** Sociodemographic factors associated with late presentation with advanced HIV disease (LP-AHD)

Variable	Total (*n* = 315)	No LP-AHD (*n* = 225)	LP-AHD (*n* = 90)	*p* value
**Age group**				0.002
18–35	41 (13.0)	32 (14.2)	9 (10.0)	
36–45	80 (25.4)	69 (30.7)	11 (12.2)	
46–55	105 (33.3)	66 (29.3)	39 (43.3)	
56–90	89 (28.3)	58 (25.8)	31 (34.4)	
**Gender**				0.140
Female	270(85.7)	197 (87.6)	73 (81.1)	
Male	45(14.3)	28 (12.4)	17 (18.9)	
**Marital Status**				0.257
Single	55(17.5)	41 (18.2)	14 (15.6)	
Divorced	61 (19.4)	44 (19.6)	17 (18.9)	
Widowed	81 (25.7)	51 (22.7)	30 (33.3)	
Married	118 (37.5)	89 (39.6)	90 (32.2)	
**Number of children**				0.290
0–1	73 (23.2)	63 (23.7)	20 (22.2)	
2–3	139 (44.3)	104 (46.4)	35 (38.9)	
4 or more	102 (32.5)	29.9 (224)	25 (38.9)	
**Educational Level**				0.480
No formal education	64 (20.3)	41 (18.2)	23 (25.6)	
Primary	63 (20.0)	44 (19.6)	19 (21.1)	
Junior High School	123 (39.0)	89 (39.6)	34 (37.8)	
Senior High School	49 (15.6)	38 (16.9)	11 (12.2)	
Tertiary Education	16 (5.1)	13 (5.8)	3 (3.3)	
**Residence**				0.067
Rural	45 (14.3)	27 (12.0)	18 (20.0)	
Urban	270 (85.7)	198 (88.0)	72 (80.0)	
**Employment Status**				0.183
Not employed	92(29.2)	59 (26.2)	33 (36.7)	
Self employed	200 (63.5)	149 (66.2)	51 (56.7)	
Employed	23 (7.3)	17 (7.6)	6 (6.7)	
**Ethnicity**				0.933
Akan	257 (81.6)	184 (81.8)	73(81.1)	
Northerners	46 (14.6)	33 (14.7)	13 (14.4)	
Others	12 (3.8)	8 (3.6)	4(4.4)	
**Religion**				0.678
Christianity	275 (87.6)	195 (86.7)	80 (89.9)	
Muslims	36 (11.5)	28 (12.4)	8 (9.0)	
Others	3 (1.0)	2 (0.9)	1 (1.1)	
**Monthly**				0.784
< 500	149 (47.5)	107 (47.8)	42 (46.7)	
500–1000	107 (34.1)	74 (33.0)	33 (36.7)	
> 1000	58 (18.5)	43 (19.2)	15 (16.7)	
**Time taken to reach clinic (Minutes)**				0.928
5–30	74 (23.6)	52 (23.3)	22 (24.4)	
31–60	141 (45.0)	102 (45.7)	39 (43.3)	
> 60	98 (31.3)	69 (30.9)	29 (32.2)	

**Note: Data presented as frequency and percentage**, ***p*-values computed by Chi-square**, ***p*** **< 0.05 and bolded means statistically significant**,** LP-AHD: Late presentation with advanced HIV disease**

### Lifestyle factors associated with late presentation with advanced HIV disease (LP-AHD)

Alcohol intake (*p* value = 0.714), alcohol Intake Frequency (*p* value = 0.152), knowledge of partner’s HIV status (*p* = 0.164), history of smoking (*p =* 0.798), action taken by participants when feeling sick (*p* = 0.167), history of Needle Sharing for Drug Use (*p* = 0.352), history of blood donation (*p* = 0.129) and number of sexual partners (*p* = 0.479) all showed no statistical significance with LP-AHD (Table 3**).**

**Table 3:** Lifestyle factors associated with LP-AHD

Variable	Total (*n* = 315)	No LP-AHD (*n* = 225)	LP-AHD (*n* = 90)	*p* value
**Alcohol intake**				0.714^a^
No	273 (88.7)	194 (86.2)	79 (87.8)	
Yes	42 (13.3)	31 (13.8)	11 (12.2)	
**Alcohol intake frequency**				0.152^b^
Daily	6 (14.3)	3 (9.7)	3 (27.3)	
Occasionally	36 (85.7)	28 (90.3)	8 (72.7)	
**History of Smoking**				0.798^b^
No	308 (98.1)	220 (98.2)	88 (97.8)	
Yes	6 (1.9)	4 (1.8)	2 (2.2)	
**Action taking when feeling sick**				0.167^a^
Heal on its own	35 (11.1)	23 (10.2)	12 (13.3)	
Buy herbal drugs	50 (15.9)	30 (13.3)	20 (22.2)	
Buy drugs from the pharmacy	62 (19.7)	47 (20.9)	15 (16.7)	
Visit the hospital	168 (53.3)	125 (55.6)	43 (47.8)	
**Number of sexual partners**				0.479^a^
0–2	109 (37.2)	79 (37.6)	30 (36.1)	
3–5	146 (49.8)	101 (48.1)	45 (54.2)	
> 5	38 (13.0)	30 (14.3)	8 (9.6)	
**History of needle sharing for drug use**				0.352^b^
no	303 (96.2)	215 (95.6)	88 (97.9)	
Yes	12 (3.8)	10 (4.4)	2 (2.2)	
**History of blood donation**				0.129^a^
No	285 (90.5)	200 (88.9)	85 (94.4)	
Yes	30 (9.5)	25 (11.1)	5 (5.6)	
**Knowledge of partner’s HIV** status				0.164^a^
No	172 (55.0)	117 (52.5)	55 (61.1)	
Yes	141 (45.0)	105 (47.5)	35 (38.9)	
**Partner’s HIV status**				0.802^a^
Negative	78 (55.3)	58 (54.7)	20 (57.1)	
Positive	63 (44.7)	48 (45.3)	15 (42.9)	

**Note: Data presented as frequency and percentage**, ^**a**^***p*-values computed by Chi-square test**, ^**b**^***p*-values computed by Fisher exact test**, ***p*** **< 0.05 and bolded means statistically significant**,** LP-AHD: Late presentation with advanced HIV disease**

### Clinical factors associated with LP-AHD

Opportunistic infections (*p* = 0.017) showed a significant association with LP-AHD. However, type of HIV infection (*p* = 0.388), history of other STDs (*p* = 0.400), opportunistic infection (*p* = 0.692), viral suppression (*p* = 0.295) and viral rebound (*p* = 0.487) did not exhibit a significant association with LP-AHD **(**Table 4**).**

**Table 4:** Clinical factors associated with LP-AHD

Variable	Total (*n* = 315)	No LP-AHD (*n* = 225)	LP-AHD (*n* = 90)	*p*-value
**Type of HIV infection**				0.388^a^
HIV 1	304 (96.8)	217 (96.9)	87 (96.7)	
HIV 2	7 (2.2)	4 (1.8)	3 (3.3)	
HIV 1 and 2	3 (1.0)	3 (1.3)	0 (0.0)	
**History of other STDs**				0.400^a^
Syphilis	21 (6.7)	18 (8.0)	3 (3.3)	
Gonorrhea	5 (1.6)	3 (1.3)	2 (2.2)	
Candidiasis	1 (0.3)	1 (0.4)	0 (0.0)	
None	288 (91.4)	203 (90.2)	85 (94.4)	
**Opportunistic Infection**				0.692^a^
No	290 (92.1)	208 (92.4)	82 (91.1)	
Yes	25 (7.9)	17 (7.6)	8 (8.9)	
**Specific Opportunistic Infections**				**0.017** ^**a**^
Candidiasis	12 (48.0)	10 (58.8)	2 (25.0)	
Tuberculosis	9 (36.0)	3 (17.6)	6 (75.0)	
Pneumonia	4 (16.0)	4 (23.5)	0 (0.0)	
**Viral Suppression**				0.295^a^
Not suppressed	70 (24.5)	47 (22.8)	23 (28.8)	
Suppressed	216 (75.5)	159 (77.2)	57 (71.3)	
**Viral Rebound**				0.487^b^
Not rebound	267 (93.4)	191 (92.7)	76 (95.0)	
Rebound	19 (6.6)	15 (7.3)	4 (5.0)	

**Note: Data presented as frequency and percentage**, ^**a**^***p*-values computed by Chi-square test**, ^**b**^***p*-values computed by Fisher exact test**, ***p*** **< 0.05 and bolded means statistically significant**,** LP-AHD: Late presentation with advanced HIV disease**

### Psychosocial factors associated with late presentation with advanced HIV disease

Various reasons for delayed testing (*p* < 0.001) showed a significant association with LP-AHD. Additionally, participants with a fear of perceived side effects of ART (*p* = 0.002), participants who expressed concerns about confidentiality (*p* = 0.002), had difficulty accessing healthcare (*p* = 0.029), fear of stigma associated with HIV/AIDS (*p* < 0.001), participants who disclosed their HIV Status to partner/close relative (*p* = 0.011), participants who sought medical care that eventually led to their HIV diagnosis (*p* < 0.0001) were all significantly associated with LP-AHD **(**Table 5**).**

**Table 5:** Psychosocial factors associated with LP-AHD

Variable	Total (*n* = 315)	No LP-AHD (*n* = 225)	LP-AHD (*n* = 90)	*p* value
**Reason for Delayed Testing**				< 0.001
I didn’t feel sick	139 (47.4)	115 (56.7)	24 (26.7)	
I wasn’t told to get test	131 (44.7)	80 (39.4)	51 (56.7)	
I preferred not to know my HIV status	6 (2.0)	1 (0.5)	5 (5.6)	
I didn’t know where to get test	7 (2.4)	5 (2.5)	2 (2.2)	
I thought the test was expensive	10 (3.4)	2 (1.0)	8 (8.9)	
**Perceived side effects of ART**				0.002
No	210 (67.5)	161 (72.9)	49 (54.4)	
Yes	101 (32.5)	60 (27.1)	41 (45.6)	
**Concerns about confidentiality**				**0.002**
No	210 (67.5)	161 (72.9)	49 (54.4)	
Yes	101 (32.5)	60 (27.1)	41 (45.6)	
**Difficulty accessing healthcare**				**0.029**
No	201 (64.8)	151 (68.6)	50 (55.6)	
Yes	109 (35.2)	69 (31.4)	40 (44.4)	
**Fear of stigma associated with HIV/AIDS**				**< 0.001**
No	190 (61.3)	149 (67.7)	41 (45.6)	
Yes	120 (38.7)	71 (32.3)	49 (54.4)	

**Note: Data presented as frequency and percentage**, ***p*-values computed by Chi-square test**, ***p*** **< 0.05 and bolded means statistically significant**,** LP-AHD: Late presentation with advanced HIV disease**

### Predictors of LP-AHD

In a univariate logistic regression model, participants aged 36–45 years (cOR: 0.30, 95% CI: (0.14–0.65), *p* = 0.002) showed a 70% reduced likelihood of LP-AHD compared to the reference age group (56–90 years). Moreover, participants who were diagnosed based on clinical suspicion (cOR: 17.36, 95% CI: (2.33–129.33), *p* = 0.005), were not tested after reporting to the hospital with symptoms (cOR: 3.06, 95% CI: (1.74–5.36), *p* < 0.0001), thought HIV testing was expensive (cOR: 19.17, 95% CI: (3.83–95.96), *p* < 0.001), had perceived severe side effects of ART (cOR: 2.39, 95% CI: (1.29–4.44), *p* = 0.006), had concerns about confidentiality (cOR: 2.25, 95% CI: (1.35–3.74), *p* = 0.002), had difficulty accessing healthcare (cOR: 1.75, 95% CI: (1.06–2.90), *p* = 0.029) or had a fear of stigma associated with HIV/AIDS (cOR: 2.51, 95% CI: (1.52–4.14), *p* < 0.001) were associated with decreased chances of presenting late with advanced HIV disease.

In a multivariate logistic regression model, participants lower aged of 36–45 years were associated with approximately 70% decreased chances of LP-AHD (aOR: 0.32, 95% CI: (0.14–0.60), *p* = 0.004). Participants who weren’t told to get tested after reporting to the hospital (aOR: 2.47, 95% CI: (1.30–4.74), *p* = 0.006), were diagnosed based on clinical suspicion hospital (aOR: 13.86, 95% CI: (1.83–104.80), *p* = 0.011) or thought HIV testing was expensive hospital (aOR: 7.04, 95% CI (1.31–37.91), *p* = 0.023) **(**Table 5**).**

**Table 6:** Predictors of LP-AHD

Variable	LP-AHD	cOR	*p*-value	aOR	*p*-value
**Age group (Years)**					
18–35	9 (10.0)	0.53 (0.22–1.24)	0.143	0.54 (0.23–1.29)	0.167
36–45	11 (12.2)	0.30 (0.14–0.65)	**0.002**	0.32 (0.14–0.69)	**0.004**
46–55	39 (43.3)	1.11 (0.61–1.99)	0.738	1.14 (0.63–2.06)	0.671
56–90	31 (34.4)	1.00	-	1.00	-
**Circumstance surrounding** **diagnosis**					
Screening to prevent mother to child transmission	1 (1.1)	1.00		1.00	
Clinical Suspicion	83 (92.2)	17.36 (2.33-129.33)	**0.005**	13.86 (1.83–104.80)	**0.011**
Routine Screening	3 (3.3)	3.84 (0.38–39.19)	0.256	4.05 (0.39–42.31)	0.242
Partner was positive	3 (3.3)	6.86 (0.66–71.81)	0.108	5.45 (0.50–59.50)	0.165
Blood screen during donation	0 (0.0)	0.00 (0.00–0.00)	1.000	0.00 (0.00–0.00)	1.000
**Reason for delayed testing**					
I didn’t feel sick	24 (26.7)	1.00	-	1.00	-
I wasn’t told to get test	51 (56.7)	3.06 (1.74–5.36)	**< 0.001**	2.47 (1.30–4.74)	**0.006**
I didn’t know where to get test	2 (2.2)	1.92 (0.35–10.47)	0.453	1.54 (0.14–12.32)	0.234
I thought the HIV care was expensive	8 (8.9)	19.17 (3.83–95.96)	**< 0.001**	7.04 (1.31–37.91)	**0.023**
**Perceived side effects of ART**					
No	49 (54.4)	1.00	-	1.00	-
Yes	41 (45.6)	2.39 (1.29–4.44)	**0.006**	2.12 (0.92–4.85)	0.080
**Concerns about confidentiality**					
No	49 (54.4)	ref	-	1.00	-
Yes	41 (45.6)	2.25 (1.35–3.74)	**0.002**	1.67 (0.79–3.52)	0.180
**Difficulty accessing healthcare**					
No	50 (55.6)	1.00	-	1.00	-
Yes	40 (44.4)	1.75 (1.06–2.90)	0.229	1.04 (0.56–1.95)	0.905
**Fear of stigma associated with** **HIV/AIDS**					
No	41 (45.6)	1.00	-	1.00	-
Yes	49 (54.4)	2.51 (1.52–4.14)	**< 0.001**	1.67 (0.80–3.46)	0.170
**Specific opportunistic infections**					
Candidiasis	2 (25.0)	1.00	**-**	1.00	-
Tuberculosis	6 (75.0)	1.19 (0.86–1.45)	0.567	1.04 (0.46–1.33)	0.512
Pneumonia	0 (0.0)	0.00 (0.00–0.00)	1.000	0.00 (0.00–0.00)	1.000

**Note: cOR: crude odds ratio**,** aOR: adjusted odds ratio**,** CI: Confidence interval**, ***p*** **< 0.05 and bolded means statistically significant**,** LP-AHD: Late presentation with advanced HIV disease**

## Discussion

Although there has been remarkable progress in HIV prevention and treatment, LP-AHD is still a major public health problem globally. A significant proportion of people living with HIV do not know they are infected and therefore seek medical care late. This study revealed that approximately 29% (28.6%) of the study participants presented late for care at WHO HIV stages 3 and 4. Moreover, the age group of 36–45 years, perceived high cost of HIV care, diagnosis based on clinical suspicion, and missed opportunities for early diagnosis by were associated with LP-AHD.

The prevalence of LP-AHD in this study, 28.6% was lower compared to previous studies conducted by Gesesew et al., in Ethiopia (65.5%) [5] and Jeong et al., in Asia (72-83.3%) [19]. The disparity could be due to the utilization of just WHO staging for defining LP-AHD in this study whilst the other studies employed both CD4 count and WHO staging in their classification. Although the prevalence of LP-AHD in this study is lower compared to the overall prevalence of late presentation in Africa estimated to be between 35% and 65%, it still undermines the 95-95-95 targets set by the UNAIDS to end HIV/AIDS by 2030.

The current study did not find any significant association between gender and LP-AHD. However, there was a larger proportion of males (37.8%) who presented with LP-AHD compared to females (27.0%); even though majority of study population included in the study were females (85.7%). This could be due to the fact that men may not be seeking care consistently and are accessing treatment at a later stage of their disease than women, which has also been reported in previous study [20]. Previous studies have cited being male as a strong correlate for LP-AHD [14, 21, 22]. However this trend conflicts with other studies which showed that being female is a risk factor for LP-AHD [5, 23]. This study findings call for enhance public health education especially among males on the clinical important of continuous health seeking behaviors.

Moreover, participants in lower age group between 36 and 45 years bracket were less likely to present late than their older counterparts and this is supported by other studies which showed that old age was a predictor of LP-AHD [13, 24]. This may be due to the misconception that older people are perceived to be at lower risk of HIV infection, which may lead to lower awareness and a lower sense of urgency for regular HIV testing in older populations.

Also, seeking herbal medication was a significant cause of two-fold increased likelihood of LP-AHD among study participants. Reliance on unorthodox medication has also been reported by Agaba et al. as a cause of LP-AHD [25]. Over dependence on herbal medicine and faith healers coupled with unaware of HIV status could account for HIV progression and eventual presentation with advanced HIV disease.

Investigating the type of HIV infection did not reveal a conclusive statistically significant relationship with LP-AHD. However, a previous study conducted in Guinea-Bissau showed that HIV-2 and HIV-1/2 dually infected patients had lower risk of late presentation compared with HIV-1 infected patients [26]. Differences in study populations and methods may account for this disparity. While history of other STDs and opportunistic infection were not statistically significant findings in this current study, results from a prospective study of 115 PDWH in Turkey [27] showed that opportunistic infections were likely implications of late diagnosis as the immune system is weakened and more susceptible to other infections. People with such infections fall into the WHO HIV staging criteria for late presentation and are therefore diagnosed as such.

Although there is routine HIV testing in the Ghanaian healthcare setting, individuals may not be screened for HIV unless they specifically request a test or present with symptoms that prompt clinicians to consider HIV testing. This finding ties in with the missed chances of diagnosis observed in this study as patients were not tested initially when they reported to the hospital with symptoms.

Furthermore, those who perceived HIV testing as expensive showed a nineteen times increased risk of LP-AHD. This calls for awareness creation as HIV testing is covered by the Ghana national health insurance [28]. Furthermore, concerns about confidentiality were associated with more than a two-fold increased risk of LP-AHD although it didn’t reach significance after adjusting for cofounders. Other studies have also identified confidentiality issues as a barrier to HIV self-testing [13, 29] showing the importance of privacy protection and adherence to ethical standards in the healthcare setting.

The findings from this study could inform policymakers, public health officials, and HIV physicians in their fight against HIV. By recognizing the challenges to a timely HIV diagnosis, physicians can prioritize HIV testing, especially for people who show evidence of symptoms or risk factors. Additionally, awareness of the association between the perceived cost of HIV treatment and delayed presentation may prompt physicians and public health officials to address financial misinformation regarding HIV care since WHO has been HIV care entirely free as well increase access to testing and treatment options.

This study presents a novel data on the prevalence and predictors of LP-AHD in Ghana. However, using WHO HIV grading alone to distinguish between late and non-late presenters limited the scope of this study. Additionally, our study used participant self-reported responses and available hospital records, which presented issues with subject bias and data completeness. The retrospective nature of this study also shows the need for a prospective investigation that would allow for real-time monitoring of late presenters and, also to reduce the possibility of bias. Future studies are needed to investigate the immunological correlates of late presenters.

## Conclusion

The prevalence of LP-AHD among people diagnosed with HIV is high. Missed opportunities for early diagnosis by clinicians, diagnosis based on clinical suspicion, old age and perceived cost of HIV care were associated with LP-AHD. These factors call for targeted campaigns among the general populace and clinicians alike to promote routine HIV testing and enhanced education on the awareness of early signs of HIV.

## Data Availability

All data generated or analyzed during this study are included in this article and can be requested from the corresponding author.

## Abbreviations

HIV/AIDS: Human Immunodeficiency Virus/ Acquired Immune Deficiency Syndrome (AIDS)
PDWH: People diagnosed with HIV
LP-AHD: Late presentation with advanced HIV/AIDS disease
CHRPE: Committee on Human Research, Publication, and Ethics
KNUST: Kwame Nkrumah University of Science and Technology
WHO: World health organization
